# Fullerol C_60_(OH)_24_ Nanoparticles Affect Secondary Metabolite Profile of Important Foodborne Mycotoxigenic Fungi In Vitro

**DOI:** 10.3390/toxins12040213

**Published:** 2020-03-27

**Authors:** Tihomir Kovač, Bojan Šarkanj, Ivana Borišev, Aleksandar Djordjevic, Danica Jović, Ante Lončarić, Jurislav Babić, Antun Jozinović, Tamara Krska, Johann Gangl, Chibundu N. Ezekiel, Michael Sulyok, Rudolf Krska

**Affiliations:** 1 Faculty of Food Technology, Josip Juraj Strossmayer University of Osijek, Franje Kuhača 20, 31000 Osijek, Croatia; bsarkanj@unin.hr (B.Š.); ante.loncaric@ptfos.hr (A.L.); jurislav.babic@ptfos.hr (J.B.); antun.jozinovic@ptfos.hr (A.J.); 2 Institute of Bioanalytics and Agro-Metabolomics, Department of Agrobiotechnology (IFA-Tulln), University of Natural Resources and Life Sciences Vienna (BOKU), Konrad Lorenzstr. 20, 3430 Tulln, Austria; tamara.krska@gmx.at (T.K.); chaugez@gmail.com (C.N.E.); michael.sulyok@boku.ac.at (M.S.); rudolf.krska@boku.ac.at (R.K.); 3 Department of Food Technology, University North, Trg dr. Žarka Dolinara 1, 48000 Koprivnica, Croatia; 4 Department of Chemistry, Biochemistry and Environmental Protection, Faculty of Sciences, University of Novi Sad, Trg Dositeja Obradovića 3, 21000 Novi Sad, Serbia; ivana.borisev@dh.uns.ac.rs (I.B.); aleksandar.djordjevic@dh.uns.ac.rs (A.D.); danica.jovic@dh.uns.ac.rs (D.J.); 5 Institute of Biotechnology in Plant Production, Department of Agrobiotechnology (IFA-Tulln), University of Natural Resources and Life Sciences Vienna (BOKU), Konrad Lorenzstr. 20, 3430 Tulln, Austria; johann.gangl@boku.ac.at; 6 Department of Microbiology, Babcock University, Ilishan Remo 121103, Ogun State, Nigeria; 7 Institute for Global Food Security, School of Biological Sciences, Queen’s University Belfast, University Road, Belfast BT7 1NN, Northern Ireland, UK

**Keywords:** fullerol C_60_(OH)_24_, nanoparticles, foodborne mycotoxigenic fungi, mycotoxins, secondary metabolism, *Aspergillus* spp., *Fusarium* spp., *Alternaria* spp., *Penicillium* spp.

## Abstract

Despite the efforts to control mycotoxin contamination worldwide, extensive contamination has been reported to occur in food and feed. The contamination is even more intense due to climate changes and different stressors. This study examined the impact of fullerol C_60_(OH)_24_ nanoparticles (FNP) (at 0, 1, 10, 100, and 1000 ng mL^−1^) on the secondary metabolite profile of the most relevant foodborne mycotoxigenic fungi from genera *Aspergillus*, *Fusarium*, *Alternaria* and *Penicillium,* during growth in vitro. Fungi were grown in liquid RPMI 1640 media for 72 h at 29 °C, and metabolites were investigated by the LC-MS/MS dilute and shoot multimycotoxin method. Exposure to FNP showed great potential in decreasing the concentrations of 35 secondary metabolites; the decreases were dependent on FNP concentration and fungal genus. These results are a relevant guide for future examination of fungi-FNP interactions in environmental conditions. The aim is to establish the exact mechanism of FNP action and determine the impact such interactions have on food and feed safety.

## 1. Introduction

The stable water-dissolved forms of fullerene C_60_ (nC_60_) are nanosized (1–100 nm). During its progression through different environmentally relevant routes, upon photoexcitation with oxygen, nC_60_ form water-soluble oxidised derivatives known as fullerols [1]. Moreover, fullerenes and fullerols are synthesised for specific applications in various industrial commodities [2,3,4,5,6]. Accordingly, the potential for fullerene (nano)materials to enter natural systems is increasing [7,8,9], which makes fullerol an environmentally relevant daughter product. Despite our efforts, their environmental reactivity, at least in the case of interaction with mycotoxigenic fungi, remains poorly defined [10,11,12,13]. 

It is common knowledge that mycotoxins are not desirable due to their worldwide negative impacts on human and animal health, economies and trade [14]. According to Eskola et al. [15], global mycotoxin prevalence is up to 60%–80%, and such a high occurrence can be explained by a combination of improved sensitivity in analytical methods, and the impact of climate change. The most relevant foodborne mycotoxin producing fungi belong the genera *Aspergillus*, *Fusarium*, *Alternaria* and *Penicillium* [16,17], and maximum levels of secondary metabolites produced by these genera are regulated by the European Union in the Commission Regulation (EC) No. 1881/2006 [18] and the Commission Recommendation 2013/165/EU [19]. Furthermore, plants and fungi can biologically modify mycotoxins by conjugation, while modifications are also possible during food processing. Today these processes contribute, at a significant rate, to food and feed contamination [16]. Accordingly, the European Food Safety Authority is trying to assess exposure and the effects on human and animal health by generating a more accurate database on the occurrence of modified mycotoxins in food and feed [20,21]. The mycotoxigenic fungal community structure has been changing due to climate change, which has put an even bigger emphasis on investigating mycotoxin occurrence. In short, mycotoxin growth and production by major foodborne fungi are highly influenced by climate change factors [22,23,24,25,26], for example, environmental temperature change is affecting contamination by aflatoxins in Eastern Europe, the Balkan Peninsula and Mediterranean regions [27]. 

Fungal secondary metabolism comprises part of their oxidative stress response pathways. Therefore, stressors to fungi from the environment, including the surrounding microbiome (biotic stressors) or drought, pH, light, neglected environmental compounds, etc. (abiotic stressors), reflect on the reactive oxygen species, and more specifically, the signals (nuclear factors) that initiate/modulate biosynthesis of secondary metabolites, if the stressor is within the level that induces adaptation and survival rather than cell death [10,28,29]. At the same time, fullerol C_60_(OH)_24_ nanoparticles (FNP) possess an antioxidative potential and poorly defined environmental reactivity [10,12,30]. These factors suggest that FNP modulation of the secondary metabolite profile of mycotoxigenic fungi would likely add to already increased contamination of the environment by mycotoxins.

The aim of this study was, therefore, to determine the effect of FNP on the secondary metabolite profile of the main foodborne mycotoxigenic fungi. It is hypothesised that FNP will modulate secondary metabolism of tested fungi, depending on the applied concentration, while an altered effect dependent on the fungal species is also expected. This study can be accepted as preliminary, as it is expected to be the driving factor for more follow-up-studies on the same issue, with results determining the direction and hypothesis of further research. It is one very important step closer to determine the exact mechanism of FNP action.

## 2. Results

### 2.1. Fullerol C_60_(OH)_24_ Nanoparticle Characterisation

**Figure 1:**
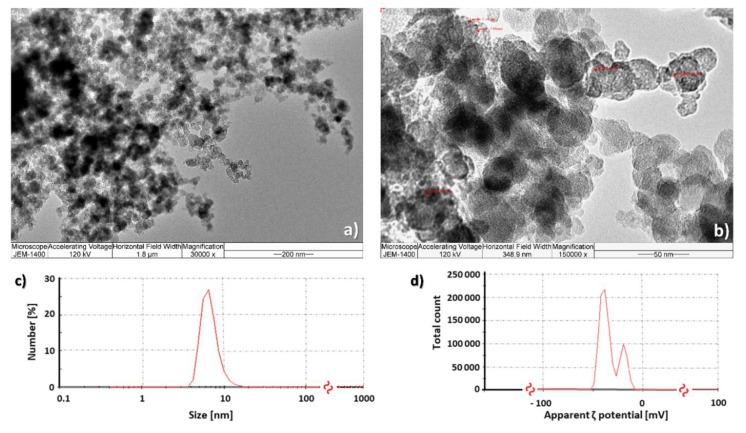
Fullerol C_60_(OH)_24_ aqueous nanoparticle solution (c = 10 mg mL^−1^) under transmission electron microscope (TEM) at 30,000× (**a**) and 150,000× (**b**) magnification, as well as particle size distribution by number (**c**) and apparent zeta potential (ζ) (**d**). The data represent one selected result out of three measurements and represent the mean hydrodynamic radius (**c**) and surface charge (**d**). *-≈-* values on *x*-axes not showed between 100–1000 on (**c**) and 0–100 on (**d**).

The results of FNP characterisation were obtained through transmission electron microscopy (TEM), dynamic light scattering (DLS) and zeta (ζ) potential measurements (Figure 1). TEM analyses showed (Figure 1a,b) that FNP aqueous solution had particles with sizes less than 10 nm as most dominant, with a tendency to form bigger agglomerates with dimensions under 100 nm. Figure 1c,d represents results of particle size distribution by the number showing that most of the FNP had the mean hydrodynamic radii in the range of 4–18 nm, having a most of the particles (27%) with a size of 7 nm (Figure 1c). The mean ζ potential value of analysed samples was −44.1 mV (Figure 1d). 

### 2.2. Impact of Fullerol C_60_(OH)_24_ Nanoparticles on the Growth of Foodborne Mycotoxigenic Fungi

**Figure 2:**
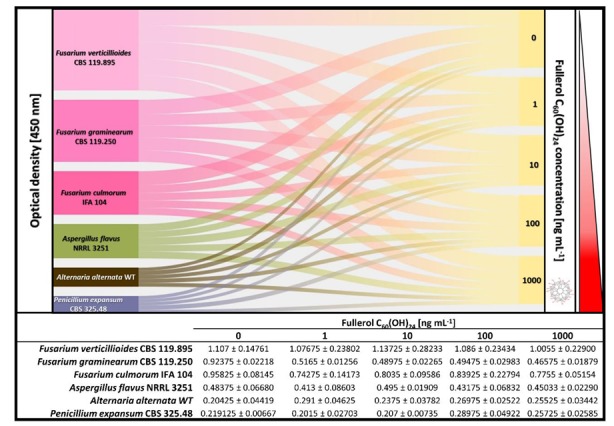
Fullerol C_60_(OH)_24_ nanoparticles (FNP) influence on ■ *Fusarium verticillioides* (CBS 119.825), ■ *Fusarium graminearum* (CBS 110.250), ■ *Fusarium culmorum* (IFA 104), ■ *Aspergillus flavus* (NRRL 3251), ■ *Alternaria alternata* (WT) and ■ *Penicillium expansum* (CBS 325.48) biomass production (expressed as optical density at 450 nm) in liquid RPMI 1640 medium incubated over a 72 h period at 29 °C. Data represent the mean ± standard error of the mean (SEM) from three separate experiments.

The FNP effect (0, 10, 100 and 1000 ng mL^−1^) on the growth of tested fungi is presented in Figure 2. After a 72 h growth period at 29 °C in RPMI 1640 media, no statistically significant difference of growth rate between treated and nontreated samples was observed, except in the case of *Fusarium graminearum* mycelia treated with the highest tested FNP dose of 1000 ng mL^−1^ (*p* = 0.03). Furthermore, a statistically significant difference in growth rate (*p* = 0.0465) was observed between FNP applied at 10 and 1000 ng mL^−1^ in the case of *Penicillium expansum*. The dose-dependent growth increase was observed in the case of *Alternaria alternata* (16% to 42%) and *P. expansum* (17% and 32% at FNP of 1 and 10 ng mL^−1^). The same dose-dependent effect, but of the opposite trend, was observed for *Aspergillus flavus* (7% to 15%), *P. expansum* (6% and 8%, only at applied FNP of 100 and 1000 ng mL^−1^) and *Fusarium* fungi, at the highest rate. *Fusarium verticillioides* growth rate decreased by 2% to 9% and *Fusarium culmorum* decreased by 12% to 22%, while *F. graminearum* growth rate was decreased at the highest rate observed in this study, from 44% to 50%. 

### 2.3. The Impact of Fullerol C_60_(OH)_24_ Nanoparticles on Secondary Metabolite Profiles of Selected Foodborne Mycotoxigenic Fungi

**Figure 3:**
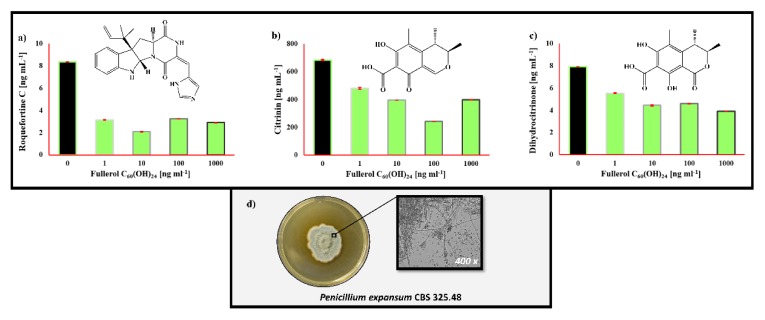
*Penicillium expansum* (CBS 325.48) secondary metabolite profile in liquid RPMI 1640 medium after 72 h at 29 °C influenced by 0, 1, 10, 100 and 1000 ng mL^−1^ of fullerol C_60_(OH)_24_ nanoparticles (FNP). Detected were (**a**) roquefortine C, (**b**) citrinin and (**c**) dihydrocitrinone. Data represent the mean ± standard error of the mean (SEM) from three separate experiments and are expressed in ng mL^−1^. *P. expansum* colony was grown on potato dextrose agar for 168 h (**d**).

**Figure 4:**
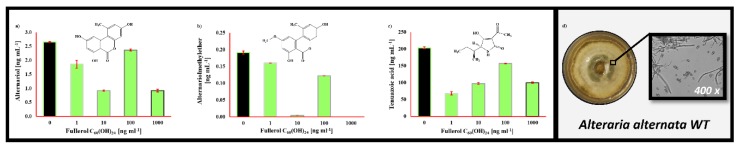
*Alternaria alternata* (WT) secondary metabolite profile in liquid RPMI 1640 medium after 72 h at 29 °C influenced by 0, 1, 10, 100 and 1000 ng mL^−1^ of fullerol C_60_(OH)_24_ nanoparticles (FNP). Detected were (**a**) alternariol, (**b**) alternariolmethylether and (**c**) tenuazonic acid. Data represent the mean ± standard error of the mean (SEM) from three separate experiments and are expressed in ng mL^−1^. *A. alternata* (WT) colony was grown on potato dextrose agar for 168 h (**d**).

**Figure 5:**
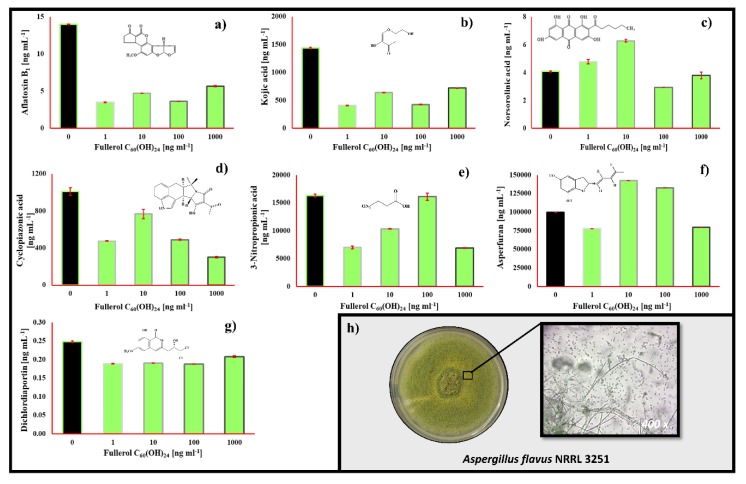
*Aspergillus flavus* (NRRL 3251) secondary metabolites profile in liquid RPMI 1640 medium after 72 h at 29 °C influenced by 0, 1, 10, 100 and 1000 ng mL^−1^ of fullerol C_60_(OH)_24_ nanoparticles (FNP) (**a**–**g**). Data represent the mean ± standard error of the mean (SEM) from three separate experiments and are expressed in ng mL^−1^. *A. flavus* NRRL 3251 colony was grown on potato dextrose agar for 168 h (**h**).

**Figure 6:**
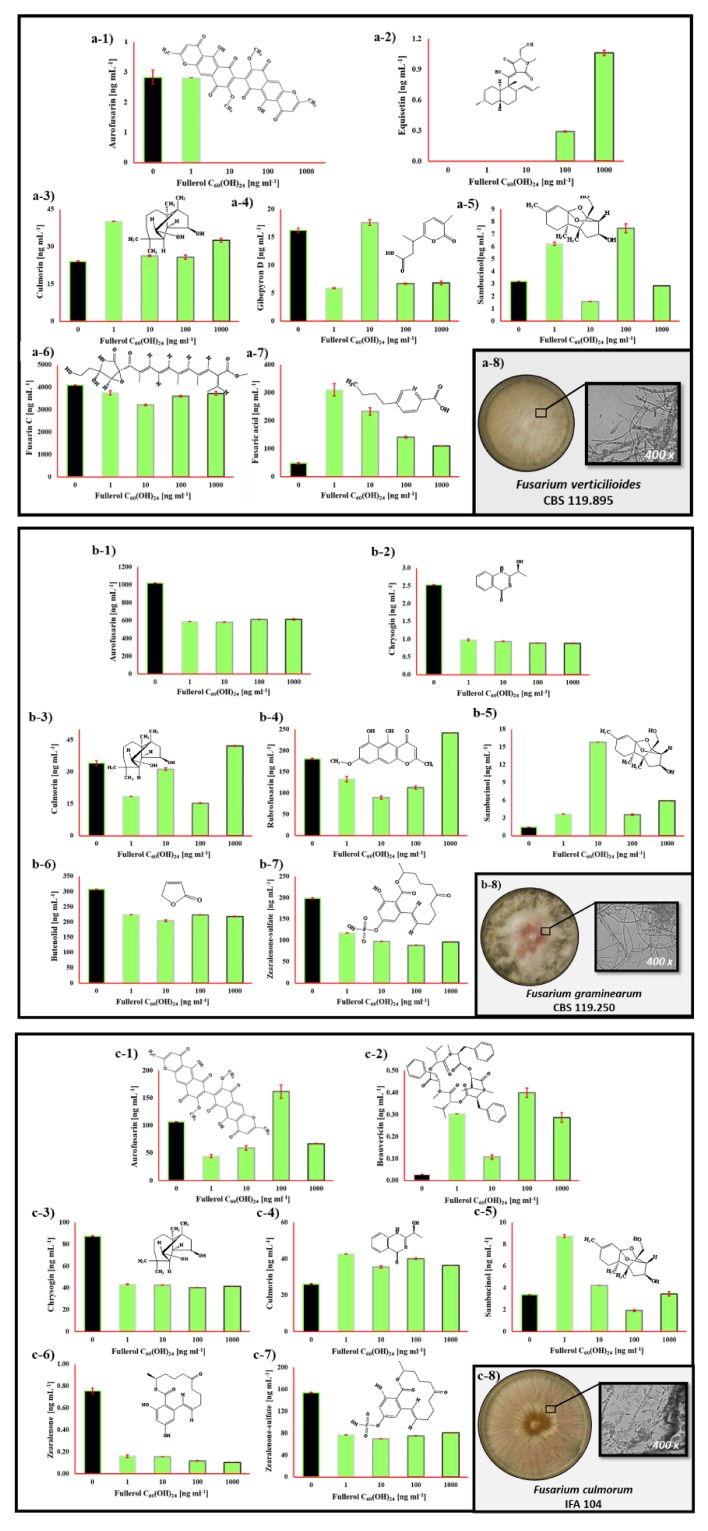
(**a**) *Fusarium verticillioides* (CBS119.825), (**b**) *Fusarium graminearum* (CBS 110.250) and (**c**) *Fusarium culmorum* (IFA 104) secondary metabolites profile in liquid RPMI 1640 medium after 72 h at 29 °C influenced by 0, 1, 10, 100 and 1000 ng mL^−1^ of fullerol C_60_(OH)_24_ nanoparticles (FNP). The detected *F. verticillioides* (CBS119.825) metabolite were (**a-1**) aurofusarin, (**a-2**) equisetin, (**a-3**) culmorin, (**a-4**) gibepyron D, (**a-5**) sambucinol, (**a-6**) fusarin C and (**a-7**) fusaric acid. *F. verticillioides* colony grown on potato dextrose agar for 168 h (**a-8**). The detected *Fusarium graminearum* (CBS 110.250) metabolites were (**b-1**) aurofusarin, (**b-2**) chrysogin, (**b-3**) culmorin, (**a-4**) rubrofusarin, (**b-5**) sambucinol, (**b-6**) butenolid and (**b-7**) zearalenone-sulfate. *F. graminearum* colony grown on potato dextrose agar for 168 h (**b-8**). The detected *F. culmorum* (IFA 104) metabolite were (**c-1**) aurofusarin, (**c-2**) beauvericin, (**c-3**) chrysogin, (**c-4**) culmorin, (**c-5**) sambucinol, (**c-6**) zearalenone and (**c-7**) zearalenone-sulfate. *F. culmorum* colony was grown on potato dextrose agar for 168 h (**c-8**). Data of all tested *Fusarium* fungi represent the mean ± standard error of the mean (SEM) from three separate experiments and are expressed in ng mL^−1^.

The FNP effect on the secondary metabolite profiles of selected foodborne mycotoxigenic fungi is presented in Figure 3, Figure 4, Figure 5 and Figure 6.

In RPMI 1640 media, *P. expansum* (Figure 3) produced the following mycotoxins: roquefortine C, citrinin and dihydrocitrinone. These compounds were observed in our control and FNP-treated samples, however, a decrease in the production of these compounds was observed after FNP treatments. For example, roquefortine C exhibited a greater decrease in concentration (61% to 75%). The concentration of produced citrinin exhibited an FNP-related decrease of 30% to 65%, caused by FNP at 100 ng mL^−1^. The dihydrocitrinone concentration decreased by 30% to 51%. There were also observed statistically significant (*p* = 0.01) differences between the control and FNP-treated samples, including the roquefortine C concentration at 10 ng mL^−1^ of FNP, the citrinin concentration at 100 ng mL^−1^ of FNP, as well as the dihydrocitrinone concentration at 1000 ng mL^−1^ of FNP.

*Alternaria alternata* secondary metabolites produced in the RPMI 1640 media under the presence of FNP are shown in Figure 4. The presence of alternariol, alternariolmethylether and tenuazonic acid were observed, both in control and FNP treated samples. Again, in the case of all mentioned metabolites, a decrease of produced concentrations was observed, after treatment of fungi with FNP during growth, in comparison with control samples. Alternariol exhibited a concentration decrease of 11%–65% upon exposure to FNP, whereas reductions for alternariolmethylether and tenuazonic acid were reduced up to 100% and 66%, respectively. Statistically significant differences between the control and FNP-treated samples were also observed, including alternariol at 10 ng mL^−1^ of FNP (*p* = 0.026), alternariolmethylether at 1000 ng mL^−1^ (*p* = 0.01) and tenuazonic acid at 1 ng mL^−1^ (*p* = 0.01).

For the *Aspergillus flavus* secondary metabolites produced under the presence of FNP, in the RPMI 1640 media, aflatoxin B1 (AFB1), kojic acid, norsorolinic acid, cyclopiazonic acid, 3-nitropropionic acid, asperfuran and dichlordiaportin were observed both in the control and FNP treated samples (Figure 5). While AFB1, kojic acid, cyclopiazonic acid, 3-nitropropionic acid and dichlordiaportin exhibited an overall decrease in concentrations if FNP was applied, norsorolinic acid and asperfuran generally exhibited an increase in concentration during FNP exposure. 

FNP decreased AFB_1_ concentration 59%–75% and decreased 3-nitropropionic acid production by up to 58% (Figure 5). The respective reductions observed for dichlordiaportin, kojic acid and cyclopiazonic acid were found to range from 16%–24%, 50%–72% and 24%–70%. The two *A. flavus* mycotoxins that exhibited highly variable FNP-related responses (decreases and increases in concentrations) were norsolorinic acid and asperfuran. Norsolorinic acid concentrations increased by 17% and 54% (at 1 and 10 ng mL^−1^ of FNP, respectively), while 100 and 1000 ng mL^−1^ of FNP decreased its concentrations by 28% and 7%, respectively. Respective asperfuran concentrations decreased by 22% and 20% (at 1 and 1000 ng mL^−1^), but they also increased by 43% and 33% at respective FNP concentrations of 10 and 100 ng mL^−1^. AFB_1_ concentration was significantly decreased at 1 ng mL^−1^ (*p* = 0.010), dichorodiaportin at 100 ng mL^−1^ (*p* = 0.014), kojic acid at 1 ng mL^−1^ (*p* = 0.010) while cyclopiazonic acid concentration was statistically significant decreased at 1000 ng mL^−1^ (*p* = 0.010) of FNP. Moreover, a statistically significant difference (*p* = 0.010) in FNP effect was observed at norsorolinic acid between 10 and 100 ng mL^−1^ of FNP. Asperfuran concentrations were statistically significant different after application of 1 and 10 ng mL^−1^ of FNP (*p* = 0.035) and 1 and 1000 ng mL^−1^ of FNP (*p* = 0.047). 

In Figure 6, the secondary metabolite profile of selected *Fusarium* spp. fungi grown in RPMI 1640 medium during 72 h at 29 °C influenced by 0, 1, 10, 100 and 1000 ng mL^−1^ of FNP is presented. Figure 6a presents the *F. verticillioides* secondary metabolites produced under the presence of FNP. In the RPMI 1640 media, the presence of aurofusarin, culmorin, fusaric acid, sambucinol, equisetin, fusarin C and gibepyron D were observed, both in the control and FNP treated samples, except equisetin. Interestingly, no major trichothecenes were detected in the RPMI 1640 medium (deoxynivalenol, nivalenol, T-2 toxin, HT-2 toxin and their derivatives), together with fumonisins. FNP did not affect aurofusarin at 1 ng mL^−1^, but they completely blocked its production, or secretion in media, at all other tested concentrations. When culmorin was present, there was a certain increase in production, from 7% to 67%, whereas the highest increase was in the presence of FNP at 1 ng mL^−1^, again. Similarly, the fusaric acid concentrations increased even more, from 128% to 542%, at the 1 ng mL^−1^ treatment with FNP. In the case of sambucinol, the 1 and 100 ng mL^−1^ treatments caused respective increases in concentration of 94% and 133%, while 10 and 1000 ng mL^−1^ caused respective concentration decreases of 50% and 11%. Equisetin was not detected in the control samples when FNP at 1 and 10 ng mL^−1^ was applied, but it was observed after addition of 100 and 1000 ng mL^−1^ of FNP in growth media. Fusarin C concentrations were decreased by FNP, from 8% to 22%, while 10 ng mL^−1^ of FNP caused a 22% decrease. The FNP caused a decrease of gibepyron D concentrations from 58% to 64%, while its concentration was also increased 8% after addition of 10 ng mL^−1^ in growth media. Furthermore, the statistically significant differences were observed between control and FNP treated samples but also between applied FNP concentrations. Differences in culmorin and fusaric acid production were statistically significant, in comparison with control samples, after the addition of 1 ng mL^−1^ of FNP (*p* = 0.01). The fusarin C concentration was statistically significant in comparison with control samples when 10 ng mL^−1^ of FNP applied (*p* = 0.01). Moreover, there was a significant difference between the effect of FNP at 10 and 100 ng mL^−1^ on sambucinol (*p* = 0.01), while in the case of gibepyron D, a significant difference was noted between 1 and 10 ng mL^−1^.

*Fusarium graminearum* secondary metabolites produced in the RPMI 1640 media under the presence of FNP (Figure 6b) were: aurofusarin, chrysogin, culmorin, rubrofusarin, sambucinol, butenolid and zearalenone-sulfate, both in the control and FNP treated samples. FNP present in growth media at all tested concentrations reduced aurofusarin concentrations by 40%–43%, while chrysogin was reduced by 61%–65%. Culmorin concentrations were decreased by 8% to 55%, while the most effective inhibition of 55% was noted when 100 ng mL^−1^ of FNP was applied. At the same time, when the 1000 ng mL^−1^ of FNP was applied, culmorin concentration in growth media was decreased by 24%. Similar to that, rubrofusarin concentration was increased by 34% after the application of 1000 ng mL^−1^ of FNP, while at all other tested FNP doses a decrease in concentration was noted, from 26%–51%; the highest decrease when 10 ng mL^−1^ of FNP was present in the growth media. Alternatively, the highest decrease in butenolid concentrations was noted at 10 ng mL^−1^ of FNP (33%), while the decrease ranged from 27%–33%, in general. The zearalenone-sulfate concentrations were decreased by 41%–55%, at the highest rate of 55% when 100 ng mL^−1^ of FNP was present in growth media. The sambucinol concentration was decreased at all tested FNP concentrations, from 145%–984%, while 10 ng mL^−1^ of FNP caused the highest decrease of 984%. As mentioned above, not only were there statistically significant differences between control and FNP treated samples, but also between applied FNP concentrations. The aurofusarin concentration after addition of 10 ng mL^−1^ of FNP in growth media was statistically significant different (*p* = 0.01) in comparison with the control sample. The sambucinol and butenolid production was statistically significant different, after addition of 10 ng mL^−1^ of FNP (*p* = 0.01), while zearalenone-sulfate concentrations were significantly different at 100 ng mL^−1^ of FNP (*p* = 0.01). The chrysogin concentration produced under the effect of 1000 ng mL^−1^ was also statistically significantly different in comparison with control samples (*p* = 0.026). Statistically significant difference between the applied FNP concentrations was noted at culmorin between 100 and 1000 ng mL^−1^ of FNP (*p* = 0.01), while at produced rubrofusarin concentrations difference was noted between 10 and 1000 ng mL^−1^ of FNP (*p* = 0.01). 

Figure 6c shows the *Fusarium culmorum* secondary metabolites produced under the presence of FNP. In the RPMI 1640 media, the presence of aurofusarin, beauvericin, chrysogin, culmorin, sambucinol, zearalenone and zearalenone- sulfate were detected, both in control and FNP treated samples. FNP reduced aurofusarin by 44% to 58% at all tested FNP concentrations, except 100 ng mL^−1,^ which increased its concentration in growth media by 52%. Chrysogin concentration was reduced by FNP from 50%–54%, zearalenone from 79% to 86% and zearalenone-sulfate from 48%–55%. On the other hand, in the case of beauvericin, FNP increased production by 228% to 1342%, while the highest increase was caused by an FNP concentration of 100 ng mL^−1^. Moreover, culmorin concentrations were also increased in FNP presence, from 36%–63%, where FNP at 1 ng mL^−1^ caused the highest increase of 63%. As mentioned above, between *F. culmorum* secondary metabolites were again observed statistically significant differences between control and FNP treated samples, as well as between applied FNP concentrations. The beauvericin concentration after addition of 100 ng mL^−1^ of FNP in growth media was significantly (*p* = 0.01) different in comparison with the control sample, while in the chrysogin and zearalenone-sulfate concentrations differences (*p* = 0.01) were observed in the case of 10 ng mL^−1^ of FNP, culmorin at 1 ng mL^−1^ of FNP (*p* = 0.01) and zearalenone at 1000 ng mL^−1^ of FNP (*p* = 0.01). Moreover, a statistically significant difference between the applied FNP concentrations was noted in the case of aurofusarin and sambucinol at 1 and 100 ng mL^−1^ of FNP present in growth media (*p* = 0.01). 

## 3. Discussion

On the basis of the previously reported FNP effect on *A. flavus* [10,12], our goal was to determine if there is a similar effect of FNP on the secondary metabolism of other important foodborne mycotoxigenic fungi. The hypothesis was that FNP would modulate secondary metabolism of tested fungi, depending on the applied concentration, while the effect will also be genus- or species-dependent. The results herein establish the foundation for follow-up studies to determine the FNP mechanism during interaction with mycotoxigenic fungi.

The size of nanoparticles, as well as the ability to form aggregates/agglomerates, are the features that determine their properties and subsequent biological activity. Thus, results of TEM, DLS and ζ potential indicate that the prepared FNP water solution is a stable polyanionic system mostly composed of clusters with sizes less than 100 nm. These results are in accordance with our previously published results and EC Recommendation for the definition of the term “nanomaterial” [10,12,31,32,33,34].

In general, our findings are in accordance with previously reported data on FNP impact on mycotoxigenic fungi biomass production. However, available data stems from experiments only conducted with *A. flavus* [10,12], and to the best of our knowledge, this is the first report on FNP impact on representatives of *Alternaria*, *Penicillium* and *Fusarium* spp. It is evident that every tested concentration of FNP yielded no strong antifungal effect. Despite the above-mentioned differences, there were certain effects of FNP on the foodborne mycotoxigenic fungi growth rate, which is in accordance with a previous study conducted by Holmes et al. [35]. Our findings suggest that his conclusions can be applied to other mycotoxigenic fungal species. Indeed, the confirmed FNP dose-dependent effect on *A. flavus* biomass in different liquid growth media extends to other tested fungal species [10,12]. Some of the inhibitors at lower concentrations can modulate secondary metabolism, but at higher concentrations, they can affect the growth of fungi. Moreover, growth inhibition cannot be directly correlated with secondary metabolism intensity, but it is important to examine both parameters. For example, the fact that growth of fungi under abiotic stressors remains practically unaffected is in accordance with results of Medina et al. [26,36] and Kovač et al. [12]. It is known that many compounds with inhibitory potential are also able to act as antioxidants, although the mode of their action is poorly understood [35]. We have shown this to also be true for FNP, which exhibit antioxidative properties. Furthermore, it is known fact that mycotoxigenic fungi are very sensitive to oxidative status perturbations, and one of their defence mechanisms involves production and export of secondary metabolites out of the cell, at least in the case of *A. flavus* [10,29]. 

*Penicillium expansum* produced secondary metabolites under the presence of FNP, as shown in Figure 3. Roquefortine C (Figure 3a) is a modified diketopiperazine, which showed bacteriostatic activity against G+ bacteria and also can interact with cytochrome p450 and interfere with RNA synthesis; this mycotoxin is also neurotoxic to cockerels [37]. Citrinin (Figure 3b) is a polyketide compound involved in the etymology of Balkan endemic nephropathy. Toxicity and genotoxicity of citrinin are related to oxidative stress or increased permeability of mitochondrial membranes. In mice and rabbits, LD_50_ is estimated at 100 mg kg^−1^ BW, while citrinin residues may occur in edible tissues and eggs after oral exposure of animals to highly contaminated feed [38,39]. Dihydrocitrinone (Figure 3c) is a secondary metabolite that originated from *Penicillium* and *Aspergillus* spp. and is related to citrinin. To be more precise, it is the less toxic metabolite of citrinin which is found in the urine and blood of rats and humans [40]. All the mentioned metabolites of *P. expansum* were detected after growth in RPMI 1640 media, with roquefortine C, citrinin and dihydrocitrinone, exhibiting a decrease in concentration after FNP was added to the growth media. 

*Alternaria alternata* secondary metabolites produced under the presence of FNP are shown in Figure 4. Alternariol, alternariolmethylether and tenuazonic acid were all detected in the growth media. Alternariol (Figure 4a) and alternariolmethylether (Figure 4b) are dibenzo-α-pyrones, and it is believed that they are mutagenic because of their in vitro genotoxic effects. It has been shown that they interfere with topoisomerases I and II, with effects that have been described as ‘poisoning’. Tenuazonic acid (Figure 4c) belongs to a group of tetramic acid derivatives and is acutely toxic (LD_50_ 81–225 mg kg^−1^ BW; mice). It is produced as a virulence factor to facilitate the colonisation of the fungus on plants since it inhibits protein biosynthesis by suppressing the release of new proteins from the ribosomes [41]. All of the mentioned *A. alternata* metabolites detected after growth in RPMI 1640 media were decreased in concentration after FNP was added to the growth media. 

*Aspergillus flavus* secondary metabolites were produced under the presence of FNP in RPMI 1640 media, as shown in Figure 5. AFB_1_, kojic acid, norsorolinic acid, cyclopiazonic acid, 3-nitropropionic acid, asperfuran and dichlordiaportin were detected in the growth media. The impact of FNP on the *A. flavus* secondary metabolite profile was already reported [10,12]. However, these experiments were conducted in different microbiological media, so these results are the first generated from *A. flavus* grown in RPMI 1640 media for this purpose, but are in still in accordance with previous reports. Again, there was observed reduction of secondary metabolite concentrations in growth media as we already stated [10,12,13], which is proof of a reasonably conducted and well-designed study. Accordingly, the detected metabolites will not be explained in detail because they can be found in recently published studies.

Secondary metabolites produced by selected *Fusarium* spp. fungi are shown in Figure 6. The results confirm that most of the secondary metabolite pathways are specific to the species [42]. All three species produced aurofusarin, culmorin and sambucinol (Figure 6a–c), equisetin, gibepyron D, fusarin C, while fusaric acid was produced only by *F. verticillioides* (Figure 6a). At the same time, chrysogin, rubrofusarin, butenolid and zearalenone-sulfate were produced by *F. graminearum* (Figure 6b), while beauvericin and zearalenone were produced by *F. culmorum* (Figure 6c). The aurofusarin is dimeric naphtho-γ-pyrone and is produced by many ascomycetes where predation and mechanical damage stimulate aurofusarin synthesis. It is a red pigment known from pure culture of the fungi (Figure 6b-8)) or from maize ears that are infected with *F. graminearum* [43,44,45]. In general, aurofusarin concentration in growth media was decreased at all isolates, except when 100 ng mL^−1^ of FNP was applied during *F. verticillioides* growth (Figure 6c-1). Furthermore, sambucinol and culmorin are increased in concentration, except in the case of culmorin produced by *F. graminearum* (Figure 6). Culmorin is an ‘emerging toxin’ with a sesquiterpene diol core structure, not so much investigated and produced by *Fusarium* spp. fungi, *F. culmorum* (name giving) for example. Its toxicological properties are poorly described in the literature, but the LD_50_ is estimated for mice from 250–1000 μg kg^−1^ BW [46]. At the moment, the role of sambucinol, a major metabolite of *F. culmorum*, is unknown. However, there is evidence that culmorin can seriously affect the deoxynivalenol metabolism in mammals by suppressing glucuronidation [47]. Equisetin is a secondary metabolite of *F. verticillioides* detected in growth media after addition of 100 and 1000 ng mL^−1^ of FNP (Figure 6a-2). It is a member of the acyl tetramic acid family of natural products. There are reports about its biological activity as an antibiotic and for its HIV inhibitory activity, cytotoxicity and mammalian DNA binding. For those reasons, it is synthesised under laboratory conditions [48]. Furthermore, the observed concentrations of gibepyron D, known for its nematocidal activity [49], were dependent upon added FNP dose (Figure 6a-4). Fusarin C, a metabolite that shows mycoestrogenic properties [50], is slightly decreased or unaffected in concentration after addition of FNP (Figure 6a-6). Fusaric acid (Figure 6a-7), picolinic acid derivative produced by *Fusarium* spp. fungi, responsible for induced programmed cell death and altered MAPK signalling in healthy PBMCs and Thp-1 cells [51], showed an increase in concertation after all tested FNP doses. The chrysogin, butenolid, rubrofusarin, zearalenone, beauvercin and zearalenone-sulfate, *F. graminearum* and *F. culmorum* metabolites, were also detected in growth media, as already mentioned. While there are no reports about chrysogin activity in the scientific literature, butenolid is reported as a myocardial mitochondria dysfunction inducer, while rubrofusarin is a pigment and an intermediate in the aurofusarin biosynthesis pathway [52]. Zearalenone-sulfate is a modified form of zearalenone, which exhibits low acute toxicity, but acts as a full and partial agonist on oestrogen receptors α and β, and cause reproduction disorders in pigs. Moreover, it is an activator of receptors involved in the regulation of cytochrome P450 isoforms in vitro. Due to that, it potentially affects the phase I metabolism of various endo- and xenobiotics [53]. Beauvericin is a nonribosomal cyclic hexadepsipeptide that possesses insecticidal properties and which can induce apoptosis of mammalian cells [54]. Beauvericin and rubrofusarin concentrations were increased by FNP, while chrysogin, butenolid, zearalenone and zearalenone-sulfate concentrations were decreased in growth media due to FNP presence (Figure 6b,c).

## 4. Conclusions

The exposure of FNP directly affected mycotoxin concentrations in important foodborne fungi, indicating that FNP definitely modulates fungal secondary metabolism. Our findings support the continued study of FNP impact on mycotoxigenic fungal species. Future studies will explore gene expression, as well as the oxidative status of fungal cells. This will allow us to confirm that the FNP effect on fungi is concentration-dependent and that due to oxidative status, modulation of the cells changes the secondary metabolite profile. This will be helpful in establishing a scientific outline on the nature of the FNP-fungi interaction, and the exact impact FNP will have on food safety.

## 5. Materials and Methods

### 5.1. Chemicals

Acetonitrile and methanol (both HPLC grade) were obtained from Merck (Darmstadt, Germany). Ammonium acetate, glacial acetic acid (p.a.), MOPS, and RPMI 1640 medium were purchased from Sigma–Aldrich (Vienna, Austria). Sodium hydroxide was purchased from Panreac (Barcelona, Spain). For ultrapure water preparation, a Purelab ultra system (ELGA LabWater, Celle, Germany) was used. Standards used in the study were prepared according to Malachova et al. [55] and were collected from various research groups, or purchased from the following commercial sources: Romer Labs^®^ Inc. (Tulln, Austria), Sigma–Aldrich (Vienna, Austria), Iris Biotech GmbH (Marktredwitz, Germany), Axxora Europe (Lausanne, Switzerland) and LGC Promochem GmbH (Wesel, Germany).

### 5.2. Fullerol C_60_(OH)_24_ Synthesis, Preparation and Characterisation of Nanoparticle Solution

Reagents used in the process of synthesis of fullerol C_60_(OH)_24_ nanoparticles (FNP) were all analytical grade (C_60_ (99.8% purity), Br_2_, NaOH and C_2_H_5_OH). FNP were synthesised in a two-step synthetic protocol. First, polybromine derivative was synthesised by the reaction of polybromination of C_60_ by Br_2_ using FeCl_3_ as a catalyst, with the aim to obtain polybrominated derivative C_60_Br_24_ as described in the paper by Djordjevic et al. [56]. The second step included the complete substitution of bromine atoms with OH groups which were achieved in alkaline media by the use of NaOH solution. This procedure was followed by repetitive rinsing of the obtained mixture with ethanol, removing the residual components, and elution with demineralised water, according to the procedure given by Mirkov et al. [57]. The final solution of FNP in water was dried under low pressure until the dark brown powder of the desired substance was obtained. For the purpose of further experiments the calculated amount of FNP powder was dissolved in demineralised water (pH = 6.5), sonicated for 15 min, after which the final solution at a concentration of 10 ppm was obtained.

In order to obtain more complete insight concerning FNP distribution, and to complement the results of DLS measurements, we performed transmission electron microscopy analyses of aqueous FNP solution. The measurements were conducted on a JEM 1400 microscope operating at an accelerating voltage 120 kV, using a horizontal field width of 173.9 nm and magnification of 300,000×. A few drops of FNP water solution sample were applied to copper grid 300 mesh, then dried at standard room temperature and measured.

DLS was used for determination of the hydrodynamic mean diameter of the particles, while zeta potential measurements were conducted for particles surface charge determination. All measurements were performed on a Zetasizer Nano ZS (Malvern Instruments Inc., Malvern, UK), at 633 nm wavelength and 173° measurement angle (backscatter detection) in water solution at room temperature. DLS measurements were conducted in triplicate, and zeta potential measurements were performed in duplicate.

### 5.3. Tested Fungal Strains, Inoculum Preparation and Cultivation

Fungi used in this study are major producers of mycotoxins and food contaminants [12,58]: *Fusarium verticillioides* (CBS119.825), *Fusarium graminearum* (CBS 110.250), *Fusarium culmorum* (IFA 104), *Aspergillus flavus* (NRRL 3251), *Alternaria alternata* (WT) and *Penicillium expansum* (CBS 325.48).

Inoculum preparation was performed, according to Jerković et al. [59]. The number of conidia in the stock suspension was adjusted on 10^6^ CFU mL^−1^ by using a Bürker-Türk counting chamber (Haemocytometer), and checked by inoculating the dilutions to PDA agar. Working 2 × conidia suspension was prepared by diluting 200 µL of stock suspension into 10 mL of RPMI 1640 medium.

Tested fungi were cultivated in the RPMI 1640 medium, buffered with 0.164 M MOPS (34.53 g L^−1^) and adjusted to pH 7.0 with (1 M) NaOH, as recommended by the National Committee for Clinical Laboratory Standards. The medium was sterilised by filtration using a 0.22 µm bottle top filter. Sterile clear polystyrene microtiter, plates (96 U-shaped wells; Ratiolab, Dreieich, Germany) were used in the microdilution test and inoculated media was amended with sterile FNP stock solution (10 mg mL^−1^) to a final concentration of 0, 1, 10, 100 and 1000 ng mL^−1^ of FNP solution.

After inoculation plates were incubated at 29 °C in an atmospheric incubator in darkness. The 72 h incubation period was needed to read plates on a microplate reader at 450 nm (Azure boisystems, Ao absorbance microplate reader, Dublin, CA, USA). Minimal inhibitory concentration for 50% cell death (MIC50) was defined as the lowest concentration reducing the optical density by 50% at 450 nm compared with growth control.

### 5.4. Determination of Fungal Secondary Metabolites in Culture Media

The metabolites produced by tested fungi in culture media were determined by the multi-analyte ‘dilute and shoot’ LC-MS/MS method of Malachova et al. [55]. For the determination of metabolites in growth media, a ten-fold dilution of 100 µL of the medium with a mixture of extraction solvent and dilution solvent (1:1, v/v) in glass vials without any pre-treatment was performed.

The screening and detection of metabolites were performed, as described by Malachova et al. [55]. At least two sSRM transitions were monitored per metabolite (quantifier and qualifier), and according to the validation guidelines, the ratio between two transitions was used as an additional identity confirmation point. Analyst 1.7.1. was used for qualitative conformation, while Multiquant 3.0.3 (SCIEX, Vienna, Austria). was used for quantification of detected analytes.

### 5.5. Statistical Analysis

All data were expressed as the mean value ± standard error of the mean (SEM) from three separate experiments. The pooled datasets were checked for normality distribution by Shapiro-Wilk test and compared by nonparametric statistics methods (Friedman ANOVA and Kendall coefficient of concordance; Kruskal-Wallis ANOVA). The programme package Statistica 13.1 (TIBCO Software Inc., Palo Alto, CA, USA) was used, and differences were considered significant when the *p*-value was <0.05. For the drawing of the Sankey diagrams Flourish studio was used (Flourish Studio, Kiln Enterprises Ltd., London, UK).
